# Electrospun Biocomposite Polycaprolactone/Collagen Tubes as Scaffolds for Neural Stem Cell Differentiation

**DOI:** 10.3390/ma3063714

**Published:** 2010-06-19

**Authors:** Joanne M. Hackett, ThucNhi T. Dang, Eve C. Tsai, Xudong Cao

**Affiliations:** 1 Ottawa Hospital Research Institute, 501 Smyth Road, Ottawa, Ontario, K1H 8L6, Canada; E-Mail: t.nhi.dang@gmail.com (T.T.D.); 2 Department of Clinical and Experimental Medicine, Linköping University, 581 85 Linköping, Sweden; 3 Ottawa Hospital Research Institute, 725 Parkdale Avenue, Ottawa, Ontario, K1Y 4E9, Canada; E-Mail: etsai@Ottawahospital.on.ca (E.C.T.); 4 Department of Chemical and Biological Engineering, University of Ottawa, 161 Louis-Pasteur, Ottawa, Ontario, K1N 6N5, Canada; E-Mail: xcao@uottawa.ca (X.C.)

**Keywords:** neurospheres, nerve tissue engineering, electrospun nanofibers, differentiation

## Abstract

Studies using cellular therapies, scaffolds, and tubular structured implants have been carried out with the goal to restore functional recovery after spinal cord injury (SCI). None of these therapeutic strategies, by themselves, have been shown to be sufficient to achieve complete restoration of function. To reverse the devastating effects of SCI, an interdisciplinary approach that combines materials science and engineering, stem cell biology, and neurosurgery is being carried out. We are currently investigating a scaffold that has the ability to deliver growth factors for the proliferation and differentiation of endogenous stem cells. Neural stem cells (NSCs) derived from mice are being used to assess the efficacy of the release of growth factors from the scaffold *in vitro.* The fabrication of the tubular implant allows a porous scaffold to be formed, which aids in the release of growth factors added to the scaffold.

## 1. Introduction 

Irreversible damage to the central nervous system (CNS) after traumatic, ischemic, or inflammatory injury results in permanent functional impairment. As there is no curative therapy, the interdisciplinary field of neural tissue engineering has emerged as a possible solution for fabricating a biological substitute that can maintain, restore, or improve neural tissue function. Multipotent neural stem cells (NSCs) isolated from fetal or adult rodent sources have now been shown to differentiate into neural cells with appropriate phenotypes. These can possibly establish connectivity within specific CNS regions after transplantation [1,2]. However, the appropriate environmental cues are needed. 

The interaction between cells and the extracellular matrix (ECM) plays an important role in regulating progenitor cell differentiation, as well as reparative and regenerative functions. For example, Schwann cells, used to improve spinal cord repair, have been shown to produce axon sprouting ECM molecules, such as laminin, fibronectin, and collagen [3]. Hence, in the treatment of traumatic spinal cord injury (SCI), there is particular interest in developing tissue engineering scaffolds using biomaterials that mimic the functionality of the ECM. These scaffolds, in turn, would aid in controlling the appropriate differentiation of NSCs transplanted into injured spinal cords to affect regeneration. 

In 1981, CNS axon regeneration was demonstrated utilizing the permissive environment of the peripheral nervous system (PNS) to construct a bridge that would facilitate axon regeneration across a spinal cord injury site [4,5]. While CNS axon regeneration was demonstrated, the ability of the regenerating axons to reenter the spinal cord, find suitable targets, form connections, and restore function has been limited [6,7,8]. These experiments demonstrated both the possibilities and the problems of the bridging concept—axons from nearby neurons regenerated into the grafts, but rarely left the PNS graft to re-enter the CNS tissue. Since then, there have been many modifications to this cellular to tissue graft strategy, many of which have employed biomimetic materials [9,10,11,12]. It is now established that an ideal SCI neuronal bridge must integrate seamlessly into spinal cord tissue, not stimulate a glial scar, and promote axon growth and encourage re-entry into the target tissue. 

Electrospinning produces nanofibrous scaffolds with high surface area to volume ratios, and fiber diameters down to the nanometer range with sufficient pores for the cell growth, proliferation, and differentiation [13,14,15]. Various degradable biomaterials are available and a broad spectrum of nanofiber-based scaffolds with different mechanical and biochemical properties are being used for tissue engineering applications. These biomimetic scaffolds can be reconstructed to provide physical and chemical cues that match the surrounding tissues, and provide support to the particular cell type required for specific tissue engineering applications [16]. In particular, poly(ε-caprolactone) (PCL) is a synthetic, biodegradable and biocompatible polymer, which has been investigated as a biomaterial for surgery and drug delivery systems [17,18], as there are no toxic effects from degradation products [19,20]. Furthermore, PCL is the most widely investigated synthetic polymer and has FDA approval in various devices for medical applications [21]. Collagen, in contrast, is a natural ECM with excellent cell adhesion properties. Therefore, the mixture synthesized by blending collagen with PCL using electrospinning will likely produce a biocomposite scaffold that will improve the biocompatibility of PCL while preserving the mechanical strength. Additionally, the mixture will provide a hydrophilic mesh with high porosity, and will have the small fiber diameters desirable for neural tissue engineering. In this study, we fabricated electrospun PCL/collagen nanofibrous scaffolds, and characterized the scaffold’s appearance by SEM and its mechanical strength. We further established the biocompatibility of the scaffolds by determining its effect on the differentiation of NSCs into three primary phenotypes.

Multipotent neuronal stem cells obtained from a murine embryonic source are a unique population of cells capable of self-renewal [22]. The NSCs produce a large number of progeny capable of differentiating into three primary phenotypes—neurons, astrocytes, and oligodendrocytes—found in the adult mammalian CNS. A defined serum-free medium supplemented with epidermal growth factor (EGF) is used to maintain the NSCs in an undifferentiated state in the form of clusters of cells (*i.e.,* neurospheres) for several culture passages [23]. When EGF is removed and serum added to the medium, the intact or dissociated neurospheres differentiate into the three primary CNS phenotypes. Interest in NSCs is mainly due to their potentials for transplantation, regeneration, and treatment of degenerative and autoimmune diseases of the nervous system [24]. Our study was aimed at the fabrication of a biomimetic nanofibrous scaffold capable of supporting the differentiation of NSCs, with a goal towards developing a tissue engineered cell–scaffold construct for transplantation after SCI to aid in regeneration.

## 2. Results and Discussion 

### 2.1. Nanofiber Fabrication and Characterization

#### 2.1.1. SEM Analysis

**Figure 1:**
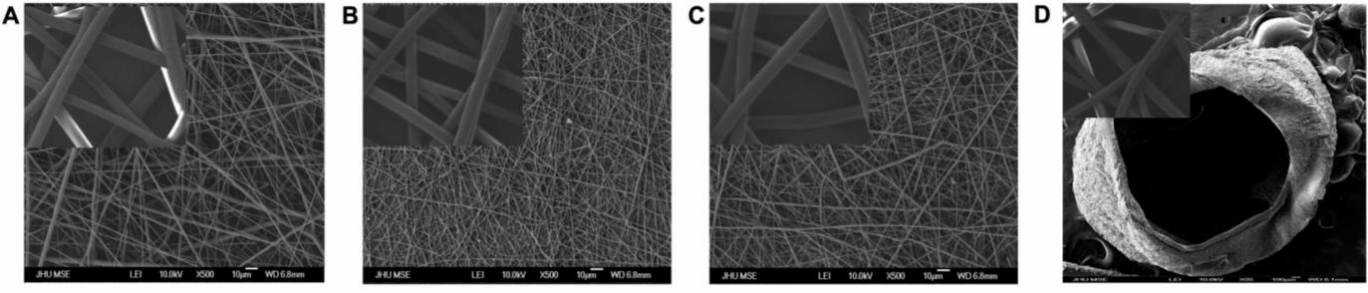
SEM images of electrospun nanofibers of (A) PCL (640 ± 83 nm), (B) collagen (330 ± 17 nm), and (C) PCL/collagen (510 ± 21 nm). PCL/collagen nanofibers weaved into a tubular scaffold are depicted in (D). Insets: Magnified fibers.

Electrospun nanofibers were characterized using SEM to reveal beadless, uniform nanofibers of PCL, collagen, and PCL/collagen with mean fiber diameters in the range of 640 ± 83 nm, 330 ± 17 nm, and 510 ± 21 nm, respectively (Figure 1). It was confirmed that both fiber diameter and alignment influenced NSC adhesion, proliferation, and differentiation (Table 1). The greatest cell adhesion was observed on the collagen fibers (diameter 387 ± 45 nm); however, NSCs proliferated and differentiated most effectively on slightly larger fibers composed of PCL/collagen (diameter 472 ± 18 nm). Aligned fibers of all treatments were favored (Table 1). 

**Table 1:** The influence of fiber diameter and alignment on adhesion, proliferation, and differentiation. Cell counts were performed three days after seeding cells on the scaffolds.

Fiber diameter (nm)	Adhesion (%)	Proliferation (%)	Differentiation (% neurons)
330 ± 17	92	84	38
510 ± 21	83	90	80
640 ± 83	68	81	58
Fiber alignment	Adhesion (%)	Proliferation (%)	Differentiation (%)
Random	73	52	56
Aligned	94	71	73

#### 2.1.2. Tensile Strength

Sufficient tensile strength is essential for a peripheral nerve substitute, as it must withstand manipulation during surgery. In addition, subsequent tissue movements associated with the cardiorespiratory cycle and patient movement must be tolerated, especially when tissue begins to infiltrate the scaffolds and axonal growth increases [25]. 

**Figure 2:**
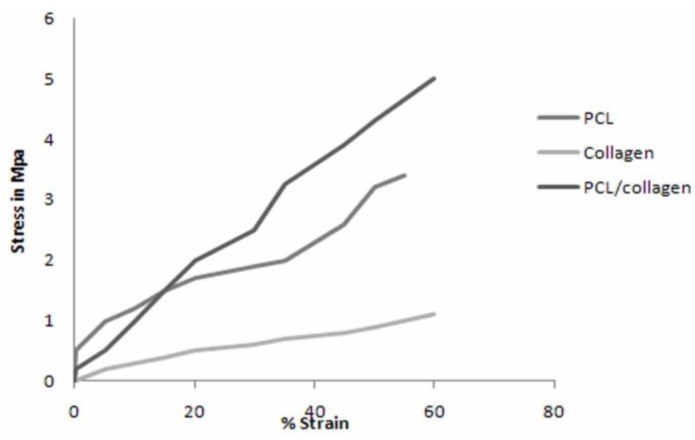
Stress-strain curve of electrospun PCL, collagen, and PCL/collagen nanofibrous scaffolds. The PCL scaffolds reached a maximum tensile strength of 3.50 MPa with an ultimate strain of 54%, the collagen scaffolds reached a maximum 1.30 MPa, but with an elongation at break of 61%, and the biocomposite PCL/collagen scaffolds had the highest tensile strength, 5.00 MPa, with a 60% elongation at break.

Figure 2 shows the maximum stress-strain curves for the electrospun PCL, collagen, and PCL/collagen nanofibers. The maximum tensile strength of the PCL scaffolds was 3.50 MPa, with an average of 1.88 ± 1.13 MPa and an ultimate strain of 54%. The electrospun collagen scaffolds had a maximum tensile strength of 1.30 MPa and an average of 0.57 ± 0.41 MPa, but with an elongation at break of 61%. The collagen scaffolds did not have sufficient tensile strength to be used as a nerve graft alone, considering that tensile strength of the fresh rat sciatic nerve is 2.72 ± 0.97 MPa [26]. When PCL and collagen were electrospun together, the nanofibrous scaffolds had a maximum and average tensile strength higher than either scaffold alone (5.00 MPa; 2.31 ± 1.71 MPa; 60% elongation at break). This biocomposite scaffold has improved tensile properties that make it suitable for neural tissue engineering.

#### 2.1.3. Degradation

**Figure 3:**
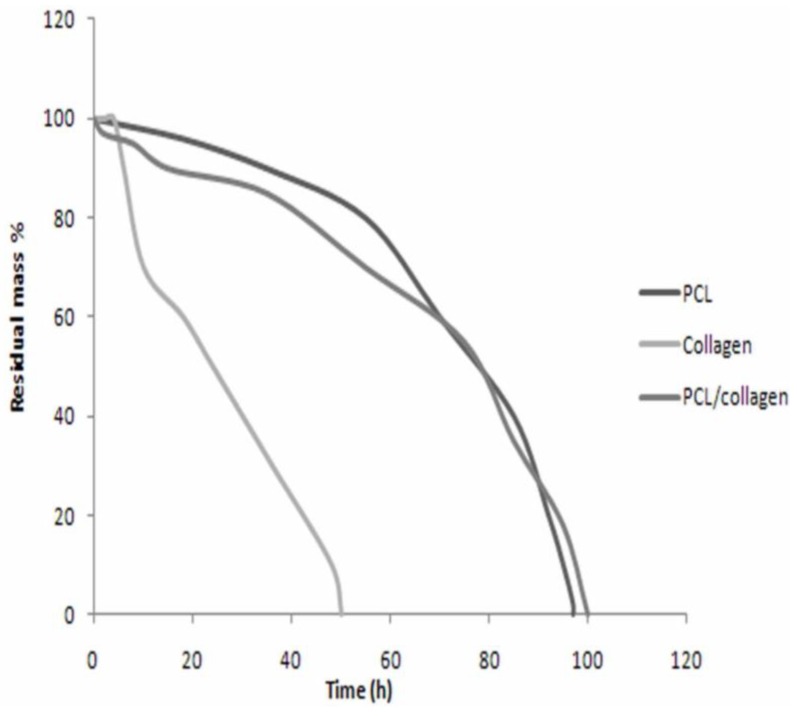
*In vitro* degradation behavior of electrospun scaffolds. PCL nanofibers resisted degradation for 96 hours, collagen nanofibers were resistant for 36 hours, and the biocomposite PCL/collagen nanofibers were resistant for 100 hours.

As shown in Figure 3, the electrospun collagen scaffold degraded faster than the PCL scaffold. When incubated in collagenase solution at 37 ^o^C for 36 hours, the collagen nanofibers were completely degraded. However, electrospun PCL scaffolds were more stable and resisted lipase degradation for 96 hours. Increased stability was observed for the biocomposite scaffold, as it took 100 hours for the complete degradation of the nanofibers in a lipase/collagenase solution. The increased stability is partially attributed to the tight interwoven mesh that forms when both PCL and collagen are mixed together. 

### 2.2. Growth Factor Release

**Figure 4:**
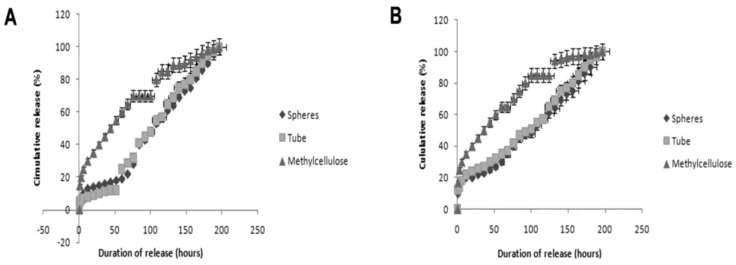
Growth factor release (%) from the various encapsulations indicated over time. (A) FGF-2, (B) NGF.

Releasing growth factors from a scaffold would be beneficial for regeneration and repair [27]. Nanofibrous scaffolds can aid in this release by having pores small enough to partially trap the growth factor inside and allow a slow release [28]. The PCL/collagen nanofibrous scaffold was tested as a delivery vehicle for fibroblast growth factor-2 (FGF-2) and nerve growth factor (NGF). These delivery capacities were compared to those of two other delivery vehicles. Only the PCL/collagen scaffold was tested, as it showed the most promise for future work, based on our goal of fabricating a scaffold that will result in small fiber diameters and high porosity, and also because of the increased biocompatibility of PCL. The growth factors were placed inside the electrospun biocomposite scaffold, or mixed with collagen or methylcellulose before being placed inside the tubular scaffold. FGF-2, NGF, collagen, and methylcellulose were chosen, as all have demonstrated the capacity to improve regeneration [29,30,31,32]. Sandwich ELISAs captured the release profile of the growth factors in different conditions. The encapsulation technique is an effective means of controlling and prolonging the release of growth factors [33]. FGF-2 shows a continuous, steady release when placed directly in the electrospun scaffold or encapsulated in collagen spheres (Figure 4A). When mixed with methylcellulose, the FGF-2 has an initial quick release, followed by a slower, steady release. Releasing FGF-2 is important for the proliferation of NSCs, especially in the oligodendrocyte lineage. As oligodendrocytes were not observed on any of the treatments (see below), release of FGF-2 may improve the differentiation of NSCs towards the oligodendrocyte phenotype.

NGF is also released slowly when placed inside the electrospun scaffold or mixed with collagen (Figure 4B). Again, there is an increased rate of NGF release when mixed with methylcellulose. As the molecular weight of both growth factors is similar, the release profiles may be due to variations in the electrospun scaffolds’ pore size. Despite the pore size inconsistencies, the PCL/collagen nanofiber scaffold can act as an effective delivery vehicle for growth factor release, thereby eliminating the need for a secondary encapsulation system to allow for a slow release profile to be achieved. The bioactivity of collected media samples, from different delivery vehicles, was assessed using neurosphere proliferation and differentiation. The PCL/collagen scaffold alone is the best delivery vehicle for enhancing proliferation under the presence of FGF-2, as the number of large primary neurospheres increased by 1.6-fold. In addition, the PCL/collagen scaffold alone is the best delivery vehicle for enhancing differentiation under the presence of NGF. There was no change in the number of large primary neurospheres observed with the PCL/collagen scaffold; however there was a 41% increase in cells that had flattened and microspike morphologies. 

### 2.3. In vitro Differentiation and Immunocytochemisty

To fabricate a neural scaffold that encourages regeneration and repair, murine NSCs were isolated and studied to gain insight into to how neurons develop and function, and also how they are affected by a variety of treatments and manipulations. These NSCs can be maintained in serum free media as neurosphere cultures for periods in excess of a year and can be successfully re-grown from liquid nitrogen storage, thus providing a long term supply [34]. Cultures can be induced to differentiate into neurons, astrocytes, and oligodendrocytes via the withdrawal of proliferative growth factors and serum addition. Once differentiated, these cells are stable and show minimal cell division over several weeks. These differentiated cells can express neuronal (e.g. Gad67) [35] and glial (e.g*.* GFAP) antigens [36], and survival can be compromised by neurotoxins [37]. Studying NSCs may therefore provide an appropriate cell model to further our understanding of basic human neural development in both health and disease.

**Figure 5:**
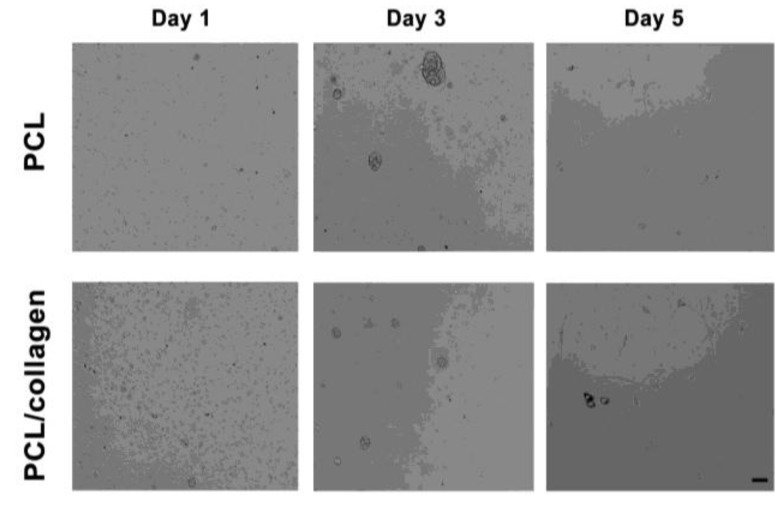
Neurospheres adhered and proliferated on electrospun PCL or PCL/collagen nanofibers after 1, 3, and 5 days. Scale bar = 100 μm.

**Figure 6:**
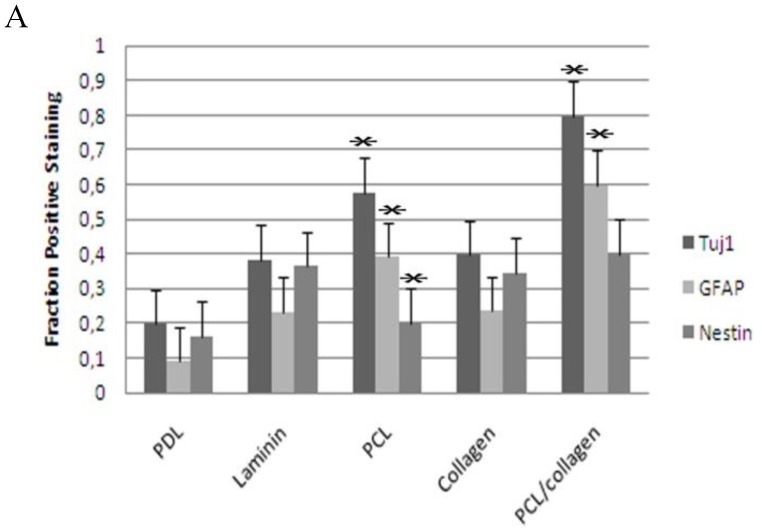

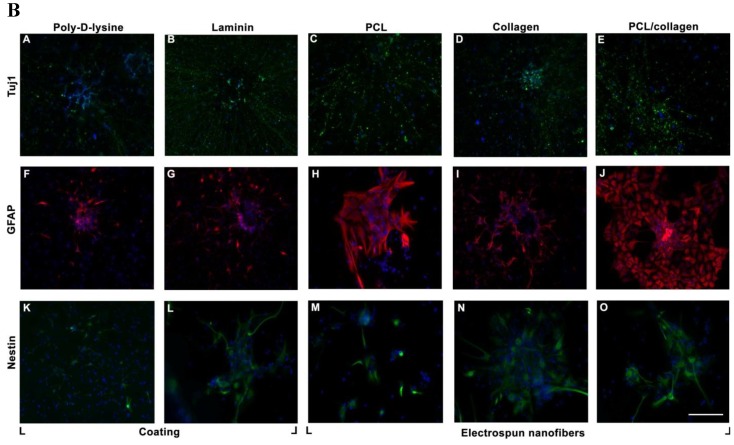
Immunofluorescence analysis of NSCs cultured on various treatments. Quantification of staining results is shown (A), with corresponding representative images of cells on each substrate (B). Representative images of cells on each substrate were stained for Tuj1 (A-E), GFAP (F-J), and Nestin (K-O). Cell nuclei (blue) were counterstained using DAPI. All cells were imaged at 20X; scale bar = 100 μm. In (A), error bars represent mean ± standard error (n = 3 different cell culture assays, greater than 2500 cells counted for each sample). *Denotes significant difference over all other samples in the same treatment (p < 0.05, ANOVA followed by Bonferroni's multiple comparison test).

*In vivo* extracellular matrices, such as collagen and laminin, exhibit micro- to nano- scale fibrous topography, which explains why electrospun matrices significantly influence the adhesion, survival, proliferation, and differentiation of stem cells [38]. Topographical features, including islands, pillars, grooves, and fibers, are observed in artificial electrospun substrates and are believed to assist in stem cell adhesion, proliferation, and differentiation in a cell-type specific manner [39]. Under proliferation conditions on Day 1, >95% of the neurospheres counted with a hemocytometer were single. These cells were seeded on electrospun PCL or PCL/collagen scaffolds to see if collagen aided in adhesion and proliferation. Cell viability, estimated by trypan blue exclusion, was around 90% for the PCL/collagen scaffold at Day 3, while it was only approximately 81% for the PCL scaffold. The small clusters observed on the PCL scaffold were dark and dense, indicating unhealthy or dead cells. By Day 5, the neurospheres on the PCL/collagen scaffolds were still mainly semi-transparent and cell viability was around 86%. Some spheres adhered to the scaffold, as the single cells were proliferating and forming small clusters of cells. The neurospheres on the PCL scaffold did not readily adhere; as the dark, dense spheres of unhealthy or dead cells lifted off, as the density of the sphere increased. Cell viability on the PCL scaffold was only around 61% by Day 5 (Figure 5). As collagen promoted proliferation on electrospun PCL, we wanted to see if it also had an effect on differentiation. To test this, neurospheres were plated as small spheres onto either poly-D-lysine (PDL), laminin coated coverslips, or electrospun meshes three days after the sixth passage, in differentiation medium and associated supplements. After seven days, the neurospheres readily adhered, flattened, and spread to yield large numbers of migrating cells. Cells were stained for neuron specific beta-Tubulin (Tuj1) to demonstrate neurons, glial fibrillary acidic protein (GFAP) for astrocytes, O4 for oligodendrocytes, and Nestin to show intermediate filament proteins to identify neuroepithelial stem cells (Figure 6). Neurons, astrocytes, and nestin-expressing cells were observed for all treatments, but oligodendrocytes were not detected. The electrospun PCL/collagen biocomposite treatment displayed the highest proportion of neurons (80%), astrocytes (60%), and Nestin positive (40%) cells. In contrast, the PDL treatment displayed the lowest proportion of neurons (20%), astrocytes (9%), and Nestin positive (16%) cells. Collagen and laminin increased the proportion of Nestin positive cells (35% and 36%, respectively), while PCL had a smaller proportion of Nestin positive cells (20%), but increased the proportion of neurons (58%) and astrocytes (39%). Similar proportions of neurons and astrocytes were observed on laminin and collagen (38% and 23%, 40% and 24%). As the electrospun PCL/collagen biocomposite scaffold generated the highest proportion of neurons, astrocytes, and Nestin positive cells, it has the potential to be an excellent scaffold for neural tissue engineering. In this cell culture model, the biocomposite scaffold increases the proportion of cells that differentiated into neurons, astrocytes, and Nestin positive cells compared to other treatments such as PDL, laminin, and collagen. The behavior of this scaffold mimics the native ECM *in vivo* [40]*,* and is therefore encouraging for use as a component of a therapeutic strategy to repair the injured spinal cord.

## 3. Experimental Section 

All chemicals were purchased from Sigma (St. Louis, MO,) and used as received, unless otherwise indicated. 

### 3.1. Electrospinning of Nanofibers

Poly(ε-caprolactone) was dissolved in 1,1,1,3,3,3-hexafluor-2-propanol (HFP), chloroform (Fisher, ON, Canada), or 80:20 dichloromethane/methanol (Fisher) to form a 15% (w/w) clear solution. Porcine collagen (Nippon Meat Packers, Tokyo, Japan) was dissolved in water/HFP at a ratio of 1:1 (v/v) and stirred overnight to form a 12.5% solution. The polymer solutions were fed separately or together into a 1 mL standard syringe attached to a 25G blunted stainless steel needle using a syringe pump (74900 Series, Cole Parmer, USA) at a flow rate of 1.0 mL/h. A high voltage of 10-12 kV (Gamma High Voltage Research, USA) was applied when the polymer solution was drawn into fibers and collected on an aluminum foil wrapped collector kept at a distance of 6 cm from the needle tip. Nanofibers were collected on 12-mm coverslips, secured along the edge with surgical glue (B-401 secure adhesive, Factor II, Lakeside, AZ), and then used for characterization and cell culture experiments. In addition, the PCL/collagen polymer solution was drawn into fibers and collected on a rotating mandrel kept at a distance of 8 cm from the needle tip. This allowed a tubular scaffold to be fabricated.

### 3.2. Morphology and Characterization of Electrospun Nanofibers

PCL, collagen, and PCL/collagen electrospun nanofibers were studied using a scanning electron microscope (SEM). Nanofibers were fixed in a mixture of 1.5% glutaraldehyde/ 3% paraformaldehyde in 100 mm sodium cacodylate buffer (pH 7.4) with 2.5% sucrose for 45 minutes at room temperature. This was followed by a fixation with 1 % osmium tetroxide in 100 mm sodium cacodylate buffer (pH 7.4) for 15 minutes at room temperature. Samples were dehydrated with a graded ethanol series (50/75/85/95/100/100 % in water) followed by hexamethyldisilizane (HMDS) or CO_2_ critical-point drying (Samdri-795, Tousimis, Rockville, MD). Subsequent 10-nm gold sputter-coating (Hummer 6.2 Sputter System, Anatech USA, Hayward, CA) allowed for imaging with environmental (FEI Quanta 200 ESEM, Hillsboro, OR) or field-emission SEM (JEOL 6700F, Tokyo, Japan). The average diameter of the electrospun fibers was analyzed from at least five different sections of the SEM images using NIH Image J software. 

Tensile properties of electrospun nanofiber scaffolds were determined using a tabletop tensile tester (Instron 3342, Canton, MA, USA) at a load cell capacity of 10 N. Dogbone shaped test specimens consisting of dimensions 5 mm breadth × 10 mm length, with a thickness of 500 μm were tested at a crosshead speed of 10 mm/min and gauge length of 20 mm, at ambient conditions [14,41]. A minimum of 10 specimens of individual scaffolds were tested until a break was endured; the results obtained were plotted for the stress–strain curve of the scaffolds. 

To determine the degradation rate of PCL, lipase was dissolved in PBS (pH 7.4) at a concentration of 7 mg/mL. The PCL samples were weighed prior placement in a tube of lipase solution kept at 37 °C. Samples were removed, blot-dried with a paper towel (until the mass remained constant), and weighed after 1, 2, 4, 7, 24 hours, and then every 24 hours, until the mass of the samples remained constant. A similar method was used to determine the degradation rate of collagen, using collagenase instead of the lipase dissolved in PBS (pH 7.4) at a concentration of 1 mg/mL [42]. For the biocomposite scaffolds, a solution containing lipase (7 mg/mL) and collagenase (1 mg/mL) was used for the degradation assay. The net weight of the scaffold was calculated by subtracting the wet chamber weight from the scaffold-containing wet chamber weight. Once the initial wet well weight was reached, a value of 0 was assigned. 

### 3.3. Growth Factor Release

Fibroblast growth factor-2 (FGF-2; VWR, ON, Canada) or nerve growth factor (NGF; Chemicon, Temecula, CA), at a concentration of 1 μg/100 μL, was released from various encapsulations to see how effective the electrospun biocomposite scaffold would be as a delivery vehicle. Growth factors were injected along the inside of the electrospun biocomposite tubular scaffold before both ends were sealed with heated tweezers. This was compared to encapsulating the growth factors inside 2% collagen or 2% methylcellulose gel (Dow, ON, Canada) and then injecting these matrices along the inside of the electrospun biocomposite tubular scaffold. The release of growth factors from the various encapsulations was monitored for 5–8 days. For each system, 100 μL aliquots of PBS solution with released growth factor was collected at 0.5 h, 1 h, 3 h, 6 h, and 12 h on the first day, followed by sampling at 8 h intervals. The concentration of released growth factor, as a function of time, was acquired using sandwich ELISAs (FGF-2: R&D Systems, MN, USA and NGF: Millipore, ON, Canada). The minimum detectable dose of each growth factor was less than 50 pg/mL. All experiments were performed in triplicate. Bioactivity was assessed by measuring proliferation or differentiation of neurospheres using a neurosphere assay [43]. FGF-2 or NGF released-media were collected and replaced with fresh media for 7 days. To assess the bioactivity of released FGF-2 or NGF, daily released and normalized (300 pg/mL) media samples (50 µL) were added to neurospheres in basal media or differentiation media in a 6-well plate (20 cells/well). The bioactivity of collected media samples, from the different delivery vehicles, was assessed on neurosphere proliferation and differentiation. Proliferation was assessed by counting the number of large primary neurospheres, whereas differentiation was determined by counting the number of flattened neurospheres with microspikes present. 

### 3.4. Tissue Preparation and Neural Stem Cell Cultures

All surgical procedures were conducted in accordance with policies established by the Canadian Council of Animal Care.

BALB/cA mouse embryos at embryonic day 13.5-14.5 (E13.5 –E14.5) were isolated after sacrifice of gravid females (Charles River, QC, Canada) and placed into ice-cold Hank's balanced salt solution (HBSS; GIBCO, ON, Canada) for extraction of neural stem cells. Retrieval of the spinal cords was collected from two to three litters of embryos at a time, and rinsed in HBSS. Following rinsing, the tissue was placed in proliferation medium (STEMCELL Technologies, BC, Canada) and mechanically dissociated by repeated gentle trituration through flamed wide bore tips (Bio-Rad, ON, Canada). The suspension was placed in a Corning T-75 flask (Corning, NY, USA) containing proliferation medium and associated supplements (STEMCELL Technologies), as well as penicillin (100 U)/streptomycin (100 μg/mL; GIBCO). Cells were grown as free-floating clusters (neurospheres) at 37 °C with 95% air and 5% CO_2_, passaged by mechanical dissociation every 5–7 days, and prevented from attachment by gently knocking the flasks every other day. Proliferation kinetics was studied by microscopic examination of cultures and by collecting neurospheres every 2 days, and assessing the total number of viable cells at each passage by Trypan Blue exclusion. For the microscopic examination, dark, dense spheres were considered to be unhealthy and composed of more dead cells than lighter colored spheres, as viable neurospheres are generally semitransparent. Initially, single cells proliferated to form small clusters of cells that lightly adhered to the scaffold; however some of these clusters lifted off as the density of the sphere increases. Cells used for transplantation or *in vitro* differentiation had been passaged five to six times.

### 3.5. In vitro Differentiation and Immunocytochemisty

Neurospheres were plated as small spheres onto poly-D-lysine (PDL) or laminin coated coverslips (Fisher), or electrospun meshes three days after the last passage, in differentiation medium and associated supplements (STEMCELL Technologies), as well as penicillin (100 U)/streptomycin (100 μg/mL; GIBCO). The cells were differentiated for seven days and then fixed for 10 minutes in 4% paraformaldehyde (PFA) at room temperature. Following rinses in PBS (pH 7) and block in a blocking solution of 5% normal goat serum and 0.25% Triton X-100 in 0.02 M PBS (PBS^+^), the cultures underwent immunocytochemistry with reaction to primary antibodies (see below) overnight at 4 °C. After 3 rinses in PBS^+^, they were further incubated in the dark with Alexa Fluor 488- and 594- conjugated secondary antibodies (1:100; Invitrogen, ON, Canada) for 2 hours at room temperature in PBS^+^. After 3 rinses in PBS, 4',6-diamidino-2-phenylindole (DAPI) was added for 5 minutes before rinsing gently with PBS and placing the coverslip on a microscope slide (Fisher). Negative controls with omission of primary antibodies were performed in parallel, and no positive signals were detected. Cells were also evaluated after 1, 3, 5, 10, 14, 21, and 28 days. Images of these cells were not included in the manuscript, as the early time points were not informative and the later time points resulted in fuzzy images due to the scaffold degradation. The same cell type trend was observed in the later time points; however because of the difficulty in visualizing the cells due to the degradation, we could not be as certain with respect to the exact numbers.

### 3.6. Antibodies 

The primary antibodies used were: Nestin (1:50; Chemicon); Tuj-1 (1:1000; Abcam Limited, UK); glial fibrillary acidic protein (GFAP, 1:100; DAKO, Denmark); and O4 (1:50; Sigma).

Secondary antibodies were Alexa Fluor 488- and 594-conjugated, either detecting mouse or rabbit IgG (1:100; Invitrogen). Images were documented using a Zeiss inverted microscope, and processed using Axiovision software (Zeiss, Hallbergmoos, Germany).

### 3.7. Statistical Analysis

Unless otherwise stated, the data presented are expressed as mean ± standard deviation (SD) of the mean. Differences between culture conditions were assessed using one-way analysis of variance (ANOVA) followed by Bonferroni’s multiple comparison test. The value of the variance P was <0.05, meaning statistical significance was accepted at the 95% confidence level.

## 4. Conclusions 

This work provides evidence that a combination of biochemical and topographical cues can influence the direction of cellular differentiation, and raises important questions regarding fate-specification mechanisms enhanced by substrate topography. Electrospun nanofibrous scaffolds provide mechanical stability, structural guidance, and a matrix for cell integration with surrounding tissue. Collagen physically supports cells by providing specific ligands for cell adhesion, thereby acting as an ECM-mimicking nano-scaffold. The electrospun PCL/collagen biocomposite scaffold can promote cell differentiation *in vitro*, similar to how the native ECM does *in vivo.* The porous nature of the electrospun scaffold can facilitate the release of growth factors to the injured spinal cord area. We found reduced fiber diameters, along with improved mechanical properties for PCL/collagen nanofibers, suggesting the scaffold’s potential application in neural tissue engineering. This scaffold can influence cell fate, and is porous enough to add growth factors, thereby allowing a proportion of desired cell types to be controlled for possible therapeutic purposes.
